# Transdermal Diagnosis of Malaria Using Vapor Nanobubbles

**DOI:** 10.3201/eid2202.151203

**Published:** 2016-02

**Authors:** Maria Rebelo, Rita Grenho, Agnes Orban, Thomas Hänscheid

**Affiliations:** Instituto de Medicina Molecular, Lisbon, Portugal (M. Rebelo, R. Grenho, T. Hänscheid);; Budapest University of Technology and Economics, Budapest, Hungary (A. Orban);; MTA-BME Lendulet Magneto-optical Spectroscopy Research Group, Budapest (A. Orban)

**Keywords:** malaria, bloodless, hemozoin, haemozoin, nanobubble, laser, noninvasive, parasites, vector-borne infections, diagnosis, *Anopheles* spp., *Plasmodium falciparum*, malaria pigment

**To the Editor:** Establishing reliable noninvasive methods for diagnosis of malaria has been a challenge. Lukianova-Helb et al. should be applauded for developing such a method on the basis of hemozoin (Hz) detection (*1*). The authors reported a proof of principle and are preparing for “large-scale studies in humans” (*2*). Such large endeavors should be based on firm evidence, so it is surprising that the results presented were from a single patient, remarkable for the unusual quadruple drug treatment (*2*). In such a scenario, to compensate for the limited data, the results should be of convincing scientific quality.

However, the case described raises several doubts that could have been addressed, such as the reliability of the diagnosis if only a thin film and a rapid test were used (co-infection excluded) and why parasitemia was not determined at the time of the device test (instead of 4 hours before and 9 hours after). What developmental stages were the parasites in at the time of the evaluation (for example, already early trophozoites containing Hz or Hz-rich gametocytes)? Why was the patient not re-evaluated to find out if repeated measurements would become appropriately negative (test-of-cure)?

The methods and results used in the study contrast with the extraordinary numbers for the limit of detection (LOD): 0.0001% in human blood and 0.00034% in a rodent model (*1*,*2*). However, the LOD is a virtual, inferred parasitemia rate based on the detection of free Hz added to uninfected blood (*1*). An LOD can be obtained from serially diluted cultures or samples (*3*). In rodent models, detection of Hz tends to be much easier (*4*). Moreover, in *Plasmodium falciparum* infections, only immature forms have been observed, with little or no detectable Hz (*5*).

The prospects of a noninvasive test for malaria are exciting. However, in times of cost restraints, any diagnostic test or intervention should provide sufficiently convincing results before consideration of resource-intensive large-scale trials.
